# Respiration, Rather Than Photosynthesis, Determines Rice Yield Loss Under Moderate High-Temperature Conditions

**DOI:** 10.3389/fpls.2021.678653

**Published:** 2021-06-24

**Authors:** Guangyan Li, Tingting Chen, Baohua Feng, Shaobing Peng, Longxing Tao, Guanfu Fu

**Affiliations:** ^1^National Key Laboratory of Rice Biology, China National Rice Research Institute, Hangzhou, China; ^2^Crop Production and Physiology Center (CPPC), College of Plant Science and Technology, Huazhong Agricultural University, Wuhan, China

**Keywords:** energy utilization efficiency, photosynthesis, respiration, yield loss, smart crops breeding, high temperature

## Abstract

Photosynthesis is an important biophysical and biochemical reaction that provides food and oxygen to maintain aerobic life on earth. Recently, increasing photosynthesis has been revisited as an approach for reducing rice yield losses caused by high temperatures. We found that moderate high temperature causes less damage to photosynthesis but significantly increases respiration. In this case, the energy production efficiency is enhanced, but most of this energy is allocated to maintenance respiration, resulting in an overall decrease in the energy utilization efficiency. In this perspective, respiration, rather than photosynthesis, may be the primary contributor to yield losses in a high-temperature climate. Indeed, the dry matter weight and yield could be enhanced if the energy was mainly allocated to the growth respiration. Therefore, we proposed that engineering smart rice cultivars with a highly efficient system of energy production, allocation, and utilization could effectively solve the world food crisis under high-temperature conditions.

## Introduction

The global mean temperature has increased by about 1°C because of greenhouse gases containing CO_2_ and CH_4_ released by human economic activities (IPCC, 2014). By the end of the 21st century, the CO_2_ concentration will increase to 421–936 ppm, and the temperature is expected to rise by 1.4–5.8°C (IPCC, 2013, 2014). As reported in a previous study, the yields of wheat and rice were increased by CO_2_ enhancement, but higher temperatures reduced their grain yield (Cai et al., 2016). The increase in CO_2_ could not compensate for the negative impact on biomass and grain yield caused by higher temperatures; under the combination of elevated CO_2_ and temperature, there were about 10–12% and 17–35% decrease in the yield of wheat and rice, respectively (Cai et al., 2016). This threatens global food security in the face of a continuously growing world population (Wheeler and von Braun, 2013; Shi et al., 2017; Gong et al., 2020). The greater and more consistent crop production must be achieved against a backdrop of climatic stress that limits yields, and the higher photosynthetic efficiency is therefore required (Shi et al., 2018; Bailey-Serres et al., 2019). Several reports have shown that engineering the D1 subunit of photosystem II and RuBisCo activase to improve the photosynthesis can enhance the thermal resistance in rice and wheat without incurring a yield penalty (Chen et al., 2020; Degen et al., 2020). Therefore, Ahmad et al. (2020) have highlighted the importance of improving photosynthesis in field crops to reduce yield losses caused by high temperature. By contrast, Sinclair et al. (2019) have argued that increasing photosynthesis is unlikely to provide a solution to world food shortages. It, therefore, remains unclear whether photosynthetic engineering can enhance crop yields in a high-temperature climate.

## The Function of Respiration and Photosynthesis in Determining Yield Loss Under High-Temperature Conditions

**Figure 1:**
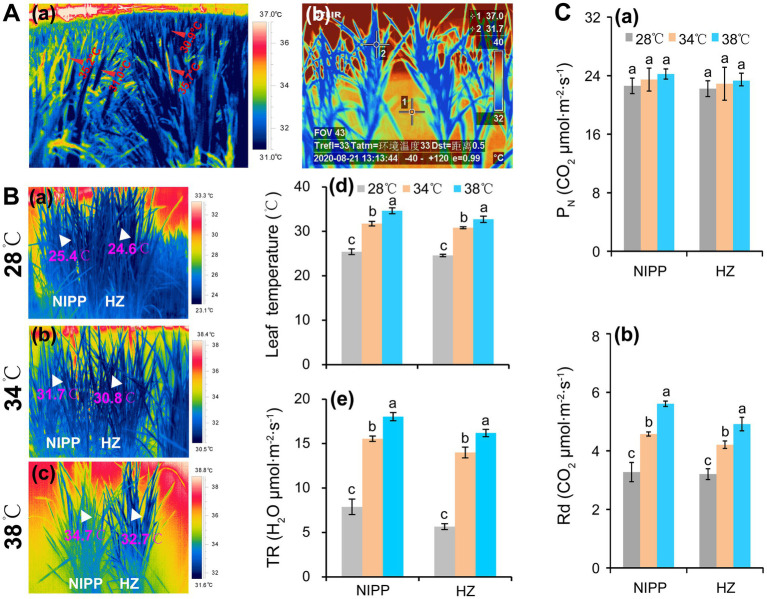
Response of rice plants to high temperatures. **(A)** Changes in leaf temperature of rice plants under high temperatures: **(a)** Panicle and leaf temperatures of rice plants grown in the paddy field under a high temperature of 37–38°C at the anthesis stage; **(b)** Leaf temperatures of rice plants grown in a greenhouse under a high temperature of 41°C at the tillering stage. **(Ba–c)** The thermal images of rice plants under 28, 34, and 38°C conditions in plant growth chambers; **(Bd)** Leaf temperature; and **(Be)** Transpiration rate (TR). **(Ca)** Net photosynthetic rate (P_N_); **(Cb)** Day respiration (Rd).

Photosynthesis would be completely inhibited in rice plants by a temperature of approximately 45°C (Crafts-Brandner and Salvucci, 2002; Li et al., 2020). When such stress lasts for more than 24 h, rice plants will die (Liu et al., 2018; Li et al., 2020). However, ambient air temperatures above 40°C typically last for only 1 or 2 h, but not the whole day. Due to intensive transpiration and morphological and phenological factors, the leaf temperatures tend to be 5–10°C lower than the ambient air temperature (Mathur et al., 2014; Fu et al., 2016; Zhang et al., 2016). In order to evaluate the temperature difference between the organ tissues (i.e., leaf and panicle) and air, a study was carried out by authors, and the air temperature was found to be 37–38°C in the field condition, while the panicle and leaf temperatures were about 35.5 and 31.0°C, respectively (Figure 1Aa). In the semi-open greenhouse, the water (1) and leaf (2) temperatures were approximately 37.0 and 31.7°C, respectively, when the air temperature reached 40–41°C (Figure 1Ab). This panicle temperature could induce spikelet sterility (Fu et al., 2016; Zhang et al., 2016), but the corresponding leaf temperature is not sufficient to inhibit photosynthesis unless it is accompanied by another abiotic stress such as high relative humidity, high light, or drought (Mathur et al., 2014; Zhang et al., 2016; Rashid et al., 2020). Also, the leaf temperature of rice plants were significantly lower than the air temperature in a plant growth chamber under different temperature conditions, which might be mainly ascribed to the intensive transpiration (Figure 1B). Accordingly, no obvious difference in net photosynthetic rate (P_N_) was shown among the temperature treatments of 28, 34, and 38°C in either rice genotypes, but the day respiration was significantly enhanced as the temperature increased (Figure 1C). It has been reported that the yield loss in rice and wheat by high night temperature is mainly ascribed to higher dark respiration, which increases the consumption of photoassimilates and thereby results in the reduction of nonstructural carbohydrates (NSC) in stem tissues (Impa et al., 2021; Xu et al., 2021). Moreover, the enhanced dark respiration restrains source availability under the combined stress of high day-and-night temperatures, leading to a considerably more severe yield penalty due to carbon loss (Xu et al., 2020). Additionally, the high midday temperature stress of 40°C caused less damage to photosynthesis but significantly decreased biomass in seagrass (George et al., 2018). Likewise, the high-temperature-tolerant wheat cultivar in Tascosa exhibited smaller reductions in biomass and lower rates of both net photosynthesis and respiration under high nighttime temperatures compared with other wheat cultivars (Impa et al., 2019). Therefore, photosynthesis might not be the main factor that leads to yield loss of rice in hot climates.

It has been reported that the average yield of four major field crops, namely, maize (*Zea mays*), wheat (*Triticum aestivum*), rice (*Oryza sativa*), and soybean (*Glycine max*), is predicted to decline by 7.4, 6.4, 3.2, and 3.1%, respectively, with every 1°C increase in the mean global temperature (Zhao et al., 2017). Interestingly, the greatest yield losses caused by the high-temperature climate were found in the C4 crop maize but not in C3 crops. This finding was inconsistent with the earlier results that C4 plants always showed high-temperature and high-light tolerance than C3 plants (Ishii et al., 1977; Berry and Björkman, 1980; Edwards and Walker, 1983). As reported by Ruiz-Vera et al. (2015), global warming resulted in a decrease in maize yield; however, the CO_2_ concentrations were enhanced by 200 ppm above the current concentration, and the photosynthetic biochemical parameters and the electron transport rate were adversely inhibited in this process. However, Rotundo et al. (2019) argued that the optimum temperature of photosynthesis in maize was approximately 40°C, rather than the common temperature-limiting functions indicating a decline in carbon assimilation above 30–33°C. This suggests possible overestimations of the negative impacts of global warming on maize yield due to the use of inadequate response functions relating carbon assimilation to temperature (Rotundo et al., 2019). In contrast, the respiration was enhanced irrespective of the enhancement of CO_2_, temperature, or in combination (Ruiz-Vera et al., 2018). Thus, the effect of global warming on maize yield loss may be due mainly to respiration rather than photosynthesis.

## Energy Production Efficiency is Enhanced in Plants Under Moderate High-Temperature Conditions

Respiration is an important biochemical process that produces ATP by oxidizing organic substrates. In annual and perennial crops, about 30–60% of the carbon assimilated during photosynthesis is lost through respiration (Cannell and Thornley, 2000). This percentage may increase with rising global temperatures according to the data presented by our group, that is, respiration is positively correlated with temperature in the physiological temperature ranging from 0 to 38°C (Figure 1Cb). A 10% yield reduction was reported in rice when the minimum nighttime temperature increased by 1°C during the growing season (Peng et al., 2004). Among winter wheat cultivars widely grown in the US Great Plains, every 1°C increase in the nighttime temperature during the seed-fill period decreased the yield by 6% (Hein et al., 2019). All these results were explained by the increased respiration at night (Sadok and Jagadish, 2020; Impa et al., 2021).

**Figure 2:**
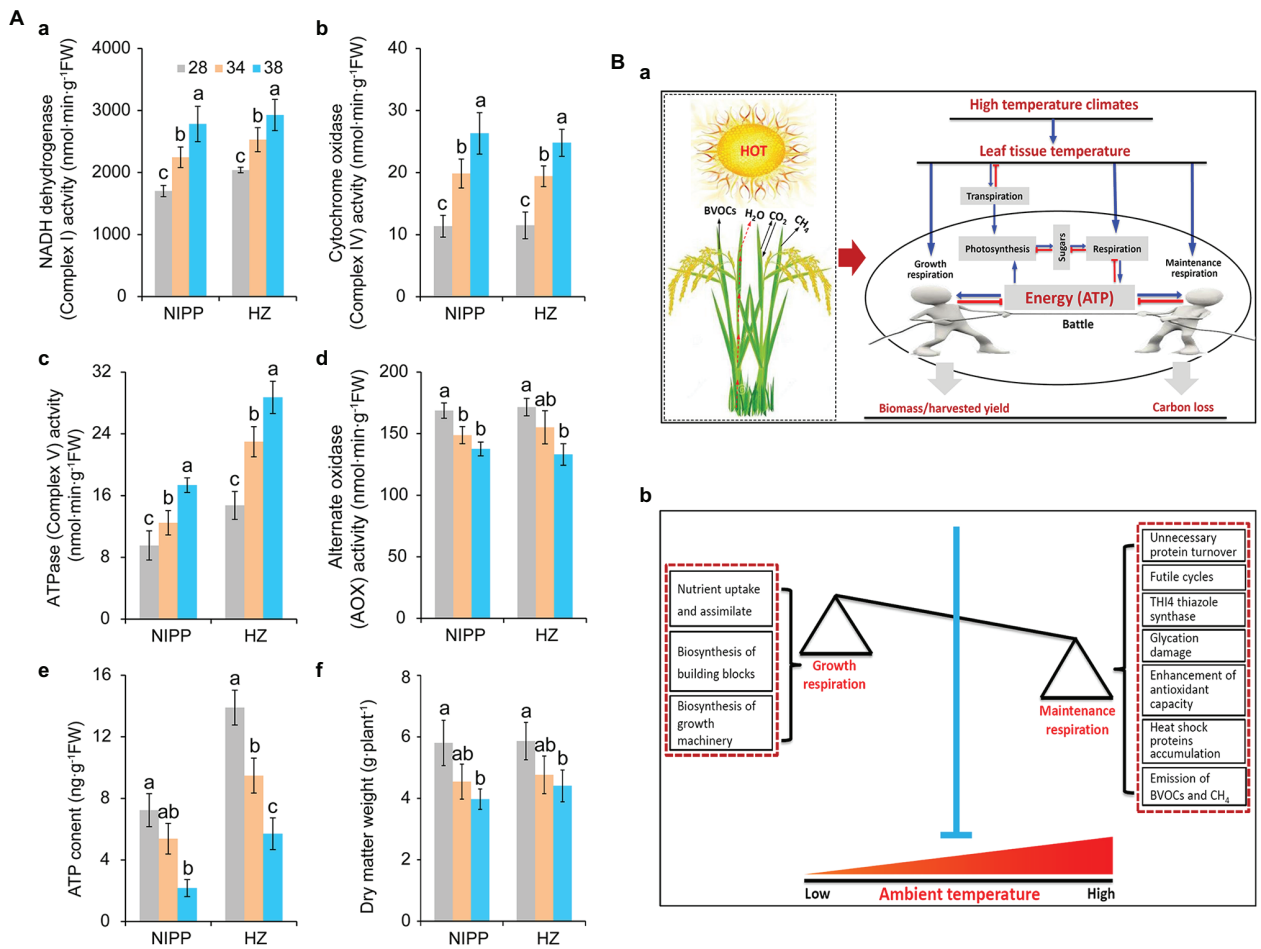
Effect of heat stress on dry weight and energy metabolism of rice plants. **(Aa)** NADH dehydrogenase; **(Ab)** Cytochrome *c* oxidase; **(Ac)** ATPase; **(Ad)** Alternative oxidase (AOX); **(Ae)** ATP content; and **(Af)** Dry weight. **(Ba)** The effect of leaf temperature on photosynthesis, respiration, and biomass of rice plants under high-temperature conditions. Leaf temperature and transpiration rate increase with increasing ambient temperature. Due to transpiration, plant leaf temperatures tend to be far below the ambient temperature, particularly under high-temperature conditions. In this case, moderate high temperature caused little damage to photosynthesis but increased respiration, requiring the consumption of more carbohydrates to produce ATP through respiration for the maintenance of biological activity. Plant biomass or harvest yield is determined by growth respiration and maintenance respiration under high-temperature conditions. **(Bb)** A model of the relationship between growth respiration and maintenance respiration in plants under global warming. The maintenance respiration processes, such as unnecessary protein turnover, futile cycling, THI4 thiazole synthase, glycation, antioxidant capacity, heat shock proteins, and even the emission of BVOCs, are activated under high temperatures. By contrast, the growth respiration processes, such as nutrient uptake and assimilation, biosynthesis of building blocks, and biosynthesis of growth machinery, are inhibited. These effects of high temperature are seen in crops with low energy utilization efficiency. Therefore, crops with high energy utilization efficiency can be engineered by inhibiting maintenance respiration processes and increasing growth respiration activities under high temperatures.

Multiprotein complexes, such as NADH dehydrogenase (Complex I), succinate dehydrogenase (Complex II), cytochrome *bc1* (Complex III), cytochrome *c* oxidase (Complex IV), ATPase (Complex V), and alternative oxidase (AOX), are involved in the respiration process and drive the cellular energy (ATP) production (Millar et al., 2011; Meyer et al., 2019). These proteins are frequently affected by abiotic stresses such as drought (Dahal et al., 2014), high temperature (Li et al., 2020; Rashid et al., 2020), cold stress (Yang et al., 2011), and high light stress (Shameer et al., 2019), thereby influencing the efficiency of ATP production. As reported earlier, respiration is controlled by the demand for ATP utilized in biosynthesis and other energy-demanding processes (Atkin and Tjoelker, 2003). Under high temperatures, rice plants exhibited higher activities of NADH dehydrogenase, cytochrome *c* oxidase, and ATPase and lower activity of AOX (Figures 2Aa–d). This result indicated that the energy production efficiency was enhanced by moderate high temperature, which might be ascribed primarily to the larger amount of energy required by the plants. Consistent with this interpretation, ATP content and dry matter weight were significantly decreased in plants under moderate high-temperature conditions (Figures 2Ae,f), suggesting that energy produced by respiration under high-temperature condition was mainly allocated to maintenance respiration rather than growth respiration (Amthor et al., 2019).

## High Energy Cost of Maintenance Respiration Processes Under Moderate High-Temperature Conditions

Respiration can be separated into two components, namely, growth respiration and maintenance respiration. In growth respiration, reduced carbon compounds are metabolized to provide energy for the addition of new biomass, whereas, in maintenance respiration, this energy is used to maintain the existing mature cells in a viable state (Amthor, 2000). Protein turnover, metabolic activity, ion transport, futile cycling, sucrose transport, and the uptake and utilization of nitrogen are enhanced in plants as temperature increases, and these processes cost large amounts of energy (Amthor et al., 2019). In fact, maintaining energy homeostasis is a challenge for all living organisms under abiotic stress conditions, and an intimate relationship exists between energy availability and stress tolerance in plants (De Block and Van Lijsebettens, 2011; Dröge-Laser and Weiste, 2018). The enhancement of antioxidant capacity and the accumulation of heat shock proteins are such energetically expensive processes that plant growth and development are inhibited, and plants may die if extreme stress lasts until an energy threshold is reached above which the damage can no longer be repaired (Baena-González and Sheen, 2008; Yu et al., 2020). Plants with reduced poly(ADP-ribose) polymerase activity consumed less NAD(H) in stressful environments and improved their energy utilization efficiency by reducing overactive mitochondrial respiration and ROS production, thereby increasing stress tolerance (Vanderauwera et al., 2007; Rissel et al., 2017; Jiang et al., 2020).

An interesting hypothesis is that plant emission of biogenic volatile organic compounds (BVOCs) and methane (CH_4_) consumes energy produced by respiration (Figure 2Ba). It has been reported that isoprene emissions are increased as the temperature enhanced in velvet bean by regulating the enzyme isoprene synthase activity (Monson et al., 1992). Similarly, the VOCs, such as acetaldehyde and (E)-2-hexenal, were released by leaves exposed to high temperatures (Loreto et al., 2006). Interestingly, these enhancements in the emission of BVOCs could confer thermal tolerance in plants and affect the atmospheric chemistry and physics that increase greenhouse gases (Peñuelas and Llusià, 2003; Peñuelas and Staudt, 2010; Kramshøj et al., 2016; Wang et al., 2019). Temperatures of 20–40°C have a strong and immediate influence on the activity of the enzymes that catalyze the synthesis of many BVOCs (Monson et al., 1992; Loreto et al., 2006; Loreto and Schnitzler, 2010). This process not only consumes energy (Niinemets et al., 1999) but also results in a large carbon loss that up to 10% of that fixed by photosynthesis under stressful conditions (Peñuelas and Llusià, 2003). Similar results were also found in the emission of CH_4_ in plants (Keppler et al., 2006; Dueck and Van Der Werf, 2008; Fraser et al., 2015; Jansson et al., 2018). Interestingly, whether in paddy fields or plant growth chambers, high-temperature-sensitive cultivars had higher rates of CH_4_ emission than high-temperature-resistant cultivars (Supplementary Figure S1). This suggests that the energy consumed in the process of CH_4_ production causes an energy deficit in the high-temperature-sensitive cultivars, thus impairing high-temperature tolerance (Figure 2Ba).

## Can the Engineering of Crops with High Energy Utilization Efficiency Reduce Yield Losses Caused by High Temperature?

Low respiration rates are generally correlated with high crop yields (Nunes-Nesi et al., 2005; Hauben et al., 2009), but very low respiration rates may not be sufficient to sustain energy production (De Block and Van Lijsebettens, 2011). Improved energy utilization efficiency is therefore immediately required to increase crop yields in a high-temperature climate. In fact, higher crop respiration does not always reduce biomass accumulation. A recent meta-analysis indicated that a high-temperature climate significantly increased biomass by 12.3% across all terrestrial plants, and this effect did not change with mean annual precipitation, experimental duration, CO_2_ enrichment, or the addition of nitrogen, drought, or irrigation (Lin et al., 2010). Similarly, grain yield was increased when plants were only subjected to high nighttime temperatures in growth chambers (Glaubitz et al., 2014). Interestingly, indica cultivars had higher respiration rates than japonica cultivars under high nighttime temperatures. Indica cultivars also showed a significant increase in biomass compared with controls, whereas japonica cultivars showed a slight decrease (Peraudeau et al., 2015). This suggested that the cultivars with increased biomass accumulation and higher respiration rates under high nighttime temperatures had greater energy utilization efficiency. More energy was allocated to growth respiration relative to maintenance respiration and even the other futile processes (Figure 2Ba). Therefore, engineering the crops with high energy efficiency in the respiration process is a feasible means of reducing yield losses caused by high temperatures.

Numerous pathways are involved in respiratory metabolism, and they are modulated by multiple genes related to all the facets of crop physiology and growth (Amthor et al., 2019). It is therefore difficult to obtain the low energy use cultivars by using conventional breeding methods. However, it is now possible to pinpoint the specific molecular targets in order to engineer greater respiratory efficiency and minimize CO_2_ loss. A number of processes have been suggested as targets for the engineering of energy-efficient crops, such as THI4 thiazole synthase activity, protection of proteins from glycation damage, nitrate acquisition, root-to-shoot nitrate assimilation, switching biosynthetic processes from nighttime to daytime, mitochondrial alternative oxidase activity, sucrose synthesis and degradation, and F6P/F16BP cycling (Amthor et al., 2019). These processes are related to energy production and consumption in plants. However, the interactions between these processes and their specific functions in the energy production and consumption of plants have not been fully characterized. Engineering the highly energy-efficient cultivars by improving these processes to increase the yields under a high-temperature climate is a worthwhile, but challenging, task.

Another promising strategy is the engineering of crops with lower BVOCs and CH_4_ emissions (Figure 2Bb). Such crops not only would have better energy utilization efficiency, reduced carbon loss, and increased yields but would also help to reduce greenhouse gas production as global warming increases. Engineering of isoprene synthesis has been reported in Cyanobacteria (Chaves and Melis, 2018), *Escherichia coli* (Liu et al., 2019; Lee et al., 2020), and *Saccharomyces cerevisiae* (Lv et al., 2016) but not in field crops.

## Engineering “Smart Crops” to Reduce Yield Losses Caused by Moderate High Temperature

Recently, Yu and Li (2021) proposed their views of breeding the so-called “smart crops,” which were defined as novel crop cultivars or even nonexisting cultivars, beyond the improved existing crop varieties. In their views, smart crops with high yield, superior quality, and high stress resistance can adapt to the climate changes rapidly by sensing the environmental signaling, nutrient, and energy status. In this case, smart crops must have a highly efficient system of energy production, utilization, and allocation to balance the growth and stress response. As analyzed earlier, the energy production efficiency is improved under higher temperature conditions, but most of which is allocated to maintenance respiration, leading to lower energy utilization efficiency (Figure 2). Therefore, energy allocation might be the most important component of smart crops, and the energy sensors of SNF1-related kinases (SnRKs) and target of rapamycin (TOR) might play a key role in this process (Dobrenel et al., 2016; Rosenberger and Chen, 2018; Crepin and Rolland, 2019). Although very few studies were conducted to reveal the relationship among SnRKs, TOR, and growth and maintenance respiration in plants under high-temperature conditions, smart crops with intelligent energy allocation are worthy of breeding to reduce the yield loss.

## Future Perspectives

The moderate high temperature causes less damage to photosynthesis but significantly increases crop respiration, and this is the main determinant of crop yield losses. The energy production efficiency is enhanced in crops under moderate high temperature, but most of this energy is allocated to maintenance respiration, decreasing energy utilization efficiency and reducing yields. In addition to protein turnover, metabolic activities, ion transport, futile cycling, sucrose transport, nitrogen uptake and utilization, antioxidant capacity, and the accumulation of heat shock proteins, plant emissions of BVOCs and CH_4_ may also consume the energy produced by respiration and release carbon fixed by photosynthesis. Engineering crops with low respiration and high energy utilization efficiency by improving these biochemical processes is both promising and challenging. The engineering of smart rice cultivars with intelligent energy allocation is seemingly an effective strategy for ensuring food security under high-temperature conditions.

The utilization of energy produced by respiration is important for crop growth and development under high temperatures; nonetheless, it has attracted relatively less attention, and there are still many outstanding questions. The interactions among the maintenance respiration processes and their functions in energy production and consumption require further characterization. The specific mechanisms by which crop plants consume energy to release BVOCs and CH_4_ must be investigated. Engineering crops with low BVOCs and CH_4_ emissions holds promise for reducing yield losses in a high-temperature climate.

## Data Availability Statement

The original contributions presented in the study are included in the article/Supplementary Material, further inquiries can be directed to the corresponding authors.

## Author Contributions

GL and TC collected the data and drafted the manuscript. BF revised the manuscript. GF, LT, and SP conceived the idea and revised the manuscript. All authors contributed to the article and approved the submitted version.

### Conflict of Interest

The authors declare that the research was conducted in the absence of any commercial or financial relationships that could be construed as a potential conflict of interest.
