# Supplementary material for: Respiration, Rather Than Photosynthesis, Determines Rice Yield Loss Under Moderate High-Temperature Conditions
Source: Front Plant Sci. 2021 Jun 24;12:678653. doi: 10.3389/fpls.2021.678653 (PMC8264589; doi:10.3389/fpls.2021.678653)
Supplement: Supplementary file 1 [file Data_Sheet_1.docx]

**Materials and methods**

**Plant materials and growth conditions**

This study was conducted at the experimental farm of the China National Rice Research Institute, Hangzhou, Zhejiang Province, China. Two rice genotypes, Nipponbare (NIPP, a japonica cultivar) and Huazhan (HZ, an indica cultivar), the seeds were directly sown in pots (20 cm height and 19 cm diameter) in a plant growth chamber with an automatic temperature control system and controlled relative humidity until the six to seven leaf stage.

At the six to seven leaf stage, NIPP and HZ plants were subjected to different temperatures, such as 28/22 °C, 34/28 °C, and 38/32 °C (day/nighttime) for 15 d, day time is from 8:30 to 16:30 and nighttime is from 16:30 to the next day 8:30. During the stress period, the relative humidity was 80/70% (day/nighttime) under natural sunlight condition. Before the end of heat stress, determined the net photosynthetic rate, respiration and plants temperature. The first fully expanded leaves were collected at the end of the heat stress period to determine mitochondrial complexes and ATP contents, and also dry matter weight. In addition, two rice cultivars were also planted in paddy field and semi-open greenhouse for temperatures determining.

**Dry matter weight measurement**

The rice plants were sampled to determine dry matter weight at the end of the heat stress. The plants were dried at 85 °C for 48 h and weighed.

**Measurement of net photosynthetic rate, respiration rate and transpiration rate**

The transpiration rate (TR) and net photosynthesis rate (P_N_) were analyzed using a Li-COR 6400 portable photosynthesis system (Li-COR Biosciences Inc., Lincoln, NE, USA) under the following conditions: photosynthetic photon flux density of 1,200 μmol·m^-2^·s^-1^; ambient CO_2_ (400 μmol·mol^-1^); 6 cm^2^ leaf area; 500 μmol·s^-1^ flow speed, and temperature according to the treatment.

The respiration rate (Rd) was analyzed by The Pn-Ci curve with a Li-COR 6400 portable photosynthesis system according to the method of Sharkey et al. (1988). The respiration rate (Rd), were estimated following the Non-rectangular hyperbola model.

**Thermal imaging of rice plants**

According to the method of Zhang et al. (2016), the temperatures of the rice plants were determined from 9:30 to 10:30 during the day using an FLIR Therma CAM™ S65 system (FLIR Systems Inc., Portland, OR. USA) with a wide-angle camera lens (18 mm IR-LENS). The camera was set up 1.0 m away from the rice plants. A black cloth was set up behind the rice plants to minimize interference from other sources when recording the temperature. The data were analyzed with Therma CAM Researcher Pro 2.7 software (FLIR Systems).

**Measurements of mitochondrial complexes and ATP content**

Mitochondrial complex Ⅰ (NADH dehydrogenase), complex Ⅳ (cytochrome c oxidase), complex Ⅴ (ATPase) and alternative oxidase (AOX) were measured with an assay kit according to the manufacturer's instructions (Comin Biotechnology Co., Ltd., Suzhou, China). The ATP contents were determined using ATP assay kits according to the manufacturer’s instructions (Geruisi Biotechnology Co., Ltd., Suzhou, China).

**The measurement of CH_4_ sampling and determine**

Two rice cultivars, Tanliangyou83 (TLY83) and Lingliangyou722 (LLY722), were planted in paddy field and pots (20 cm height and 19 cm diameter) in growth chamber. At the anthesis, TLY83 and LLY722 were subjected to 28/22 °C, 36/30 °C, and 38/32 °C (day/nighttime) for 7 d in growth chambers, and at the end of heat stress sampling the CH_4_. The static chamber method was used for sampling at 9:00 to 11:00 (Nie et al. 2020). The gas concentration was analyzed manually by gas chromatograph (Agilent 7890A, Agilent Technologies Inc, USA). The emission fluxes of CH_4_ were calculated refer to the method of Hou et al. (2016).

**Statistical analysis**

Data were processed with SPSS software 11.5 (IBM Corp., Armonk, NY, USA) to detect differences. The mean values and standard errors in the figures represent data from three experimental replicates. An ANOVA analysis was conducted to compare the difference between the different temperatures within a cultivar. Different letters indicate significant differences between the different temperatures (*P* < 0.05).

**Reference**

1. Hou H, Yang S, Wang F, Li D, Xu J (2016) Controlled irrigation mitigates the annual integrative global warming potential of methane and nitrous oxide from the rice-winter wheat rotation systems in southeast China. *Ecol. Eng.* 86:239-246. doi:10.1016/j.ecoleng.2015.11.022
2. Nie, T., Chen, P., Zhang, Z., Qi, Z., Zhao, J., Jiang, L., et al. (2020). Effects of irrigation method and rice straw incorporation on CH4 emissions of paddy fields in Northeast China. *Paddy Water Environ.* 18, 111-120. doi:10.1007/s10333-019-00768-5
3. Sharkey, T. D., Berry, J. A., Sage, R. F. (1988) Regulation of photosynthetic electron-transport in Phaseolus vulgaris L., as determined by room-temperature chlorophyll a fluorescence. *Planta* 176, 415-424. doi:10.1007/BF00395423

**Supplementary Figure legend**

**Figure S1** Effect of heat stress on the emission of CH_4_ from rice plants grown in paddy field and pots in growth chambers.
