# Supplementary figures and images for: Respiration, Rather Than Photosynthesis, Determines Rice Yield Loss Under Moderate High-Temperature Conditions
Source: Front Plant Sci. 2021 Jun 24;12:678653. doi: 10.3389/fpls.2021.678653 (PMC8264589; doi:10.3389/fpls.2021.678653)

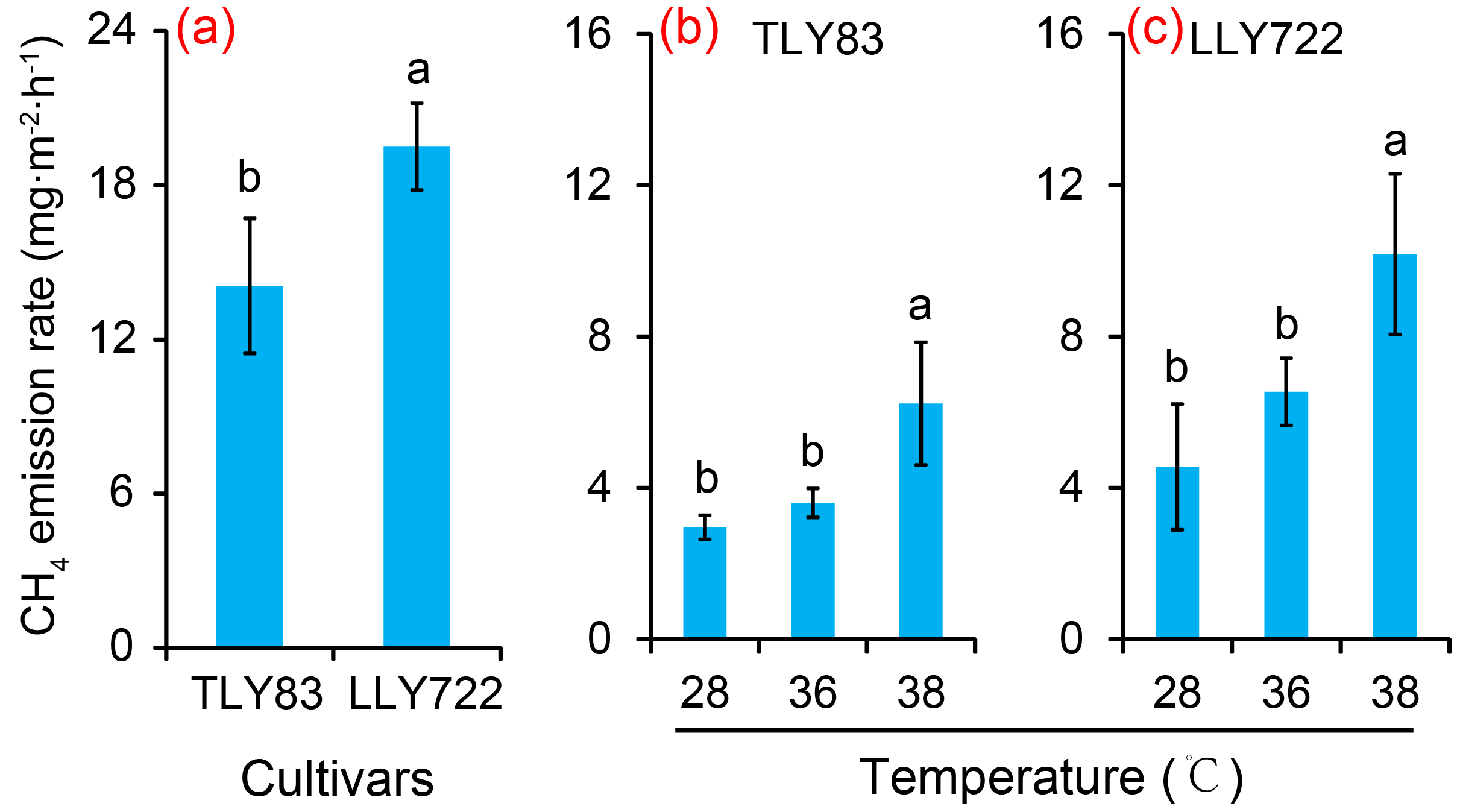

Supplement: Supplementary file 2 [file Image_1.JPEG]
